# Mapping of Phenotype Specific Host–Microbiome Protein–Protein Interaction Networks in Colorectal Cancer Using Deep Learning

**DOI:** 10.3390/ijms27104232

**Published:** 2026-05-09

**Authors:** Despoina P. Kiouri, Georgios C. Batsis, Ippokratis Messaritakis, John Souglakos, Christos T. Chasapis

**Affiliations:** 1 Laboratory of Organic Chemistry, Department of Chemistry, National and Kapodistrian University of Athens, 15772 Athens, Greece; despoina.kiouri.99@gmail.com; 2 AI Lab, Department of Digital Systems, University of Piraeus, Gr. Lampraki 126, 18534 Piraeus, Greece; gbatsis@unipi.gr; 3 Laboratory of Translational Oncology, Medical School, University of Crete, 70013 Heraklion, Greece; i_messaritakis@yahoo.com (I.M.);; 4 Laboratory of Clinical Microbiology, German Medical Institute, Yiannoukas Labs Ltd., Bioiatriki Group, Limassol 4105, Cyprus; 5 Laboratory of Pathology, University General Hospital of Heraklion, 70013 Heraklion, Greece

**Keywords:** colorectal cancer (CRC), gut microbiome, protein–protein interactions (PPI), host–microbe interactome, deep learning (DL)

## Abstract

Colorectal cancer (CRC) pathogenesis is driven by complex protein–protein interactions (PPIs) between the host and the gut microbiome, yet these molecular dialogs remain largely unmapped. This study utilizes a Deep Learning framework, enhanced by protein structure embeddings, to predict approximately 8.9 billion interspecies PPIs from clinical metagenomic data. The model achieved high accuracy with an AUROC of 0.9960, identifying a high-confidence interactome representing roughly 16% of evaluated protein pairs. Phenotype-specific analysis revealed that while microbial hubs shift—transitioning from metabolic enzymes in healthy states to transport and regulatory proteins in CRC—the primary human targets remain remarkably consistent across both cohorts. These core human interactors are predominantly metalloproteins and regulators of ubiquitination, apoptosis, and zinc transport, suggesting these pathways are primary focal points for microbial manipulation regardless of disease state. Furthermore, co-occurring bacterial genera exhibit over 99% overlap in host target profiles, indicating significant functional redundancy in microbial engagement with the host. These findings suggest that CRC probably arises from network-level perturbations of stable host signaling hubs, offering a blueprint for identifying novel therapeutic targets and biomarkers.

## 1. Introduction

Hippocrates, the father of medicine, famously stated that “All disease begins in the gut,” a prescient observation that reflects the centrality of intestinal health to systemic well-being [1]. Over the past two decades, advances in sequencing technologies have underscored the importance of the gut microbiome (GM), a densely populated microbial ecosystem comprising bacteria, viruses, fungi, archaea, and protozoa [2]. Among these, bacteria have been most extensively characterized, with human microbiome projects identifying over 2000 species spanning multiple phyla, including *Firmicutes*, *Bacteroidetes*, *Actinobacteria*, *Proteobacteria*, and *Verrucomicrobia* [3]. These organisms play critical roles in nutrient metabolism, immune system development, and epithelial barrier function, earning the microbiome the designation of a “super-organ” [4,5,6,7].

The balance of microbial populations is crucial for maintaining homeostasis. Factors such as pH, bile acids, oxygen tension, and host immune responses shape microbial communities along the gastrointestinal tract [8]. When this balance is disrupted, a state of dysbiosis arises, which has been associated with numerous conditions, ranging from inflammatory bowel disease and irritable bowel syndrome [9] to metabolic disorders [10], cardiovascular diseases [11], and autoimmune conditions [12]. Dysbiosis also affects the gut–brain axis, a bidirectional communication system linking the enteric and central nervous systems, and has been implicated in neurodevelopmental [13] and psychiatric conditions [14] such as autism spectrum disorder, major depression, and Parkinson’s disease [15,16]. These diverse links highlight the microbiome’s wide-reaching influence across human physiology and pathology.

Within this broader landscape, colorectal cancer (CRC) has emerged as one of the most compelling examples of a disease with direct microbial involvement. CRC is the second most common cancer and the third leading cause of cancer-related death worldwide [17]. The rising incidence of early-onset CRC, diagnosed before the age of 50, further emphasizes the urgency of understanding non-genetic drivers of disease. Importantly, more than 80% of CRC cases are sporadic and arise from complex interactions between host genetics, environmental factors, and the gut microbiome rather than inherited mutations [18]. The microbiome is therefore positioned not only as a marker of CRC risk but also as a potential mediator of disease onset and progression.

Specific bacterial taxa have been consistently associated with CRC. Pathogens such as *Helicobacter pylori* [19], *Salmonella* [18], and *Campylobacter jejuni* [20] contribute to carcinogenesis via chronic inflammation, toxin production, and genotoxic effects. Other taxa, including *Bacteroides fragilis*, *Escherichia coli*, and *Streptococcus gallolyticus* [21], participate in biofilm formation and secrete metabolites that modulate epithelial signaling pathways [22]. *Fusobacterium nucleatum* [23] and *Porphyromonas gingivalis*, in particular, have been linked to chemoresistance in CRC, underscoring the clinical impact of microbial activity on treatment outcomes [24]. Collectively, these findings suggest that CRC is not merely influenced by microbial imbalance but may in fact be driven by direct microbial contributions.

The molecular mechanisms that underpin these associations are increasingly recognized as pivotal for advancing both diagnostics and therapy. In recent years, research has shifted from taxonomic descriptions to mechanistic investigations at the molecular level. Protein–protein interactions (PPIs) between microbial proteins and host proteins represent one such mechanism. These interactions can alter host signaling networks, immune responses, and cellular processes in ways that favor tumor initiation or progression. While experimental approaches such as yeast two-hybrid assays, cross-linking mass spectrometry, and photo-reactive probes have shed light on microbe–host interactions [25,26,27,28,29,30,31], the scale of experimental mapping remains limited, leaving large portions of the interactome unexplored. A critical limitation in our current understanding is the paucity of experimental evidence detailing protein interactions between gut bacteria and the human host. While general human–bacterial interaction data exist in public repositories, the experimentally determined pan-human–bacterial protein interactome consists of fewer than 20,000 interactions. This limited scope, especially when contrasted with the 300–500 bacterial species estimated to inhabit the human gut, creates a research deficit that likely obstructs a deeper comprehension of how GM–host imbalances drive disease, highlighting that these molecular dialogs are largely unmapped.

Consequently, computational methods—including domain–domain interaction modeling and machine learning algorithms—have become indispensable for predicting host–microbe PPIs [32,33,34,35,36].

In this context, this work introduces a computational approach to map these crucial molecular dialogs. Next-Generation Sequencing (NGS) was applied to meticulously profile bacterial strain abundances within the gut microbiomes of both healthy individuals and colorectal cancer patients. Building on this detailed community characterization, we then applied a novel Deep Learning (DL) algorithm specifically designed to PPIs between proteins from the identified bacterial strains and proteins of the human host’s gut. The objective of this interdisciplinary study is to uncover potential protein interaction networks that could explain how specific bacterial strains contribute to, or protect against, colorectal cancer development through direct molecular engagement with the host.

## 2. Results

### 2.1. Model Re-Training Results on Test PPIs

The training procedure followed the same optimization strategy and early stopping protocol as our previous framework [37], with convergence achieved at the thirteenth epoch. The updated model, incorporating ProstT5-based embeddings, was evaluated on a held-out test set of 3,338,020 protein pairs (204,564 positive; 3,133,456 negative). Despite the pronounced class imbalance, the model demonstrated strong generalization and classification performance.

**Figure 1:**
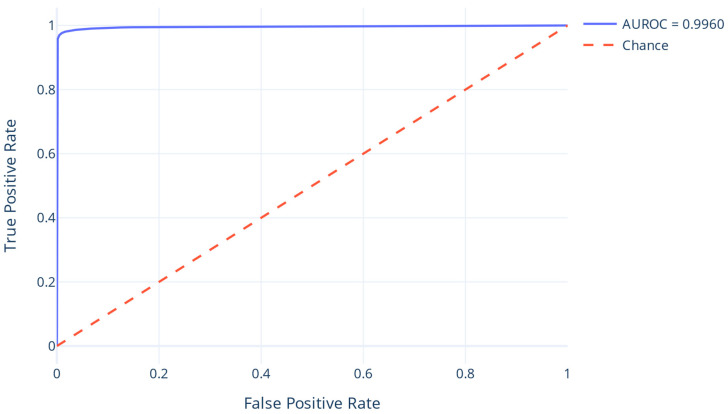
ROC curve for the test portion of the PPI dataset.

Evaluation metrics (Table 1) confirm high overall accuracy, with a macro-averaged F1-score of 0.9828 and strong performance on the interacting class (F1 = 0.9676). Imbalance-aware metrics, including a Matthews Correlation Coefficient of 0.9657 and AUROC of 0.9960 (Figure 1), further attest to the model’s robustness. The confusion matrix (Table 2) shows minimal false positive and false negative rates, while the recall at a minimum precision threshold of 0.5 reached 0.9902, validating the model’s utility for high-confidence interaction prediction.

### 2.2. Host–Microbiome PPI Network

The re-trained DL model was applied to systematically predict PPIs between human and bacterial proteins across all identified gut bacterial species from the clinical data. Using the microbiome profiles derived from the study samples, 904,554 bacterial proteins were paired exhaustively with a reference set of 9864 human proteins. This yielded a total of approximately 8.9 billion potential interspecies protein pairs. Interaction probabilities were estimated for each pair using the calibrated prediction model. To ensure high confidence in reported interactions, we applied a posterior probability threshold of 1.0. After filtering, a total of 1,481,068,857 interactions were retained as high-confidence predictions, representing approximately 16% of the evaluated protein pairs. These interactions constitute the inferred host–microbiome interactome used in downstream analyses. Detailed examples of PPIs involving the highest-degree human and bacterial proteins are documented in Supplementary Files S7 and S8, respectively.

### 2.3. Microbial Phenotype Association with Disease State

To investigate the relationship between microbial genera and disease status, bacterial genera were systematically assigned to CRC-associated, health-associated, or unassigned (“Both”) categories based on their differential abundance between healthy and CRC cohorts. Out of the 204 evaluated genera, 48 were classified as CRC-associated, 40 as health-associated, and 116 showed no clear association (Table 3). These phenotype labels were subsequently used to stratify predicted PPIs by microbial origin.

CRC-associated data exhibited significant enrichment in the CRC cohort with genera such as Peptoniphilus, Finegoldia, Porphyromonas, and Fusobacterium demonstrating highly significant q-values (e.g., Peptoniphilus: q = 2.38 × 10^−37^) (Table 4). 

Conversely, health-associated genera included well-known beneficial taxa such as *Faecalibacterium* (q = 2.91 × 10^−13^), *Subdoligranulum* (q = 1.94 × 10^−9^), and *Bifidobacterium* (q = 4.97 × 10^−5^). These genera were significantly enriched in healthy individuals (Table 5).

### 2.4. Phenotype-Specific Networks

To investigate the distinct interspecies PPI landscapes associated with health and disease, the predicted PPI network was stratified based on bacterial phenotype assignment. Specifically, two separate subnetworks were constructed: one for Health, and another for CRC-associated bacteria, by filtering interactions where each bacterial protein was annotated with a corresponding phenotype.

The Health-associated network comprised 1,258,444,460 predicted PPIs, while the CRC-associated network included 1,087,290,486 interactions. Subsequently, the proteins were also ranked by degree centrality (i.e., the number of unique interaction partners) to identify the most central host and microbial proteins in each phenotype-specific context. Results are presented in Table 6 and Table 7 for health and CRC-associated proteins, accordingly. The complete table with all the host and bacterial proteins participating in the predicted networks of both disease states can be found in Supplementary Materials File S1.

### 2.5. Analytical Protocol for the Evaluation of Molecular Network Architectures

To investigate the alignment between microbial co-occurrence and functional interactions with the human host, a comparative analysis integrating co-occurrence patterns with DL-based interspecies PPI predictions was performed. Bacterial genera participating in the same co-occurrence sub-network were cross-mapped to their corresponding host protein predicted interactors.

The overlap in host interaction profiles among bacterial pairs was assessed by computing the proportion of shared human protein interactors. A total of 126 CRC-associated bacterial pairs were analyzed. The distribution of human host interaction overlap revealed a bimodal pattern: most pairs shared >99% of human targets, while a minority showed no overlap. The mean percentage of shared targets was 84.9% (±34.8%), with a median of 99.08%.

In the health-associated cohort, 196 bacterial pairs were examined. The overlap distribution mirrored the CRC pattern, with an average similarity of 84.9% (±34.7%) and a median of 99.05% shared targets. The co-occurrence files of the Healthy-associated and Bacterial-associated cohorts can be found in Supplementary Materials Files S2 and S3.

## 3. Discussion

Beyond colorectal cancer, dysbiosis of the gut microbiome has been implicated in a wide spectrum of human diseases. Altered microbial populations have been associated with neurodevelopmental and psychiatric conditions such as autism spectrum disorder, attention deficit hyperactivity disorder, depression, Alzheimer’s, and Parkinson’s disease [15,16]. Dysbiotic microbiota have also been linked to metabolic disorders, including obesity, type 2 diabetes, and non-alcoholic fatty liver disease [38,39], as well as cardiovascular diseases like atherosclerosis and hypertension [40]. Additionally, autoimmune diseases such as rheumatoid arthritis, multiple sclerosis, and type 1 diabetes have shown connections with gut microbial imbalances [12,41].

Among the various diseases influenced by the gut microbiome, colorectal cancer (CRC) stands out due to the particularly strong and consistent evidence linking microbial dysbiosis to its pathogenesis. A key characteristic of the CRC gut microbiome is its altered composition of bacterial strains relative to healthy individuals. Until now, the bacterium that has been mostly associated with the development of CRC is *Helicobacter pylori*. In line with its role as a potent pathogen, individuals infected with *Helicobacter pylori* harbor a nearly twofold increased risk to develop CRC [19]. Several large-scale epidemiological studies in the Netherlands strongly indicate that *Salmonella* infection elevates the risk of CRC. Key findings include a standardized incidence ratio (SIR 1.54) of early-onset CRC in the proximal colon following *Salmonella* exposure, a sustained higher risk associated with *non-Enteritis* or *Typhimurium* serovars and increased serological markers of *Salmonella* exposure (FliC antibodies) in CRC patients [18]. Furthermore, inflammation resulting from *Salmonella* colonization may be a contributing factor to this increased CRC risk [18]. Another bacterial strain that has been linked to CRC is *Campylobacter jejuni*, which produces a DNA-altering cytolethal distending toxin (CDT) [20]. Although it is known that CDT contributes to the development of inflammation in the GI tract, it was recently demonstrated that not only the production of cdtB advances CRC and promotes metastasis, but also that even the presence of this type of bacteria can affect the components and transcriptional activity of the gut microbial population [20,42]. A study conducted in Taiwan that analyzed pyogenic liver abscesses (PLA), an early sign of CRC, caused by *Klebsiella pneumoniae*, revealed that those strain-specific abscesses resulted in a far greater rate of subsequent CRC than abscesses derived from other bacteria [43]. *Clostridium difficile* is another bacterium that is implicated in gastrointestinal infections and antibiotic-associated colitis. *C. difficile* produces three different toxins, two of which (i.e., toxin A (TcdA), toxin B (TcdB)) cause detrimental effects to the GI tract’s epithelial barrier, damage the cells’ genetic material as well as activate STAT3 and NF-κΒ chronic inflammation-related pathways, potentially triggering CRC pathogenesis [44]. Even though *Bacteroides fragilis* and *Escherichia coli* are a normal part of the enteric microbiome, some pathogenic strains of them have been linked to CRC [45]. Toxin-expressing strains of *B. fragilis* (*Bacteroides fragilis* toxin (bft)) and *E. coli* (colibactin (clbB)) have not only been spatially associated in biofilms in the gut, but also their synergistic pro-carcinogenic involvement in CRC has emerged [22]. A recent study by Ding et. al. revealed that *B. fragilis* promotes chemoresistance in CRC, and at the same time, phage elimination experiments conducted in mice uncovered restored chemosensitivity of those CRC cells [46]. A great number of studies have connected CRC pathology with *B. fragilis*, but its exact role remains unclear [47,48,49]. *Streptococcus gallolyticus* is an opportunistic pathogen that has been associated with numerous studies with stimulation of cell reproduction and augmentation of tumor burden [21]. Additionally, *Fusobacterium nucleatum* also promotes chemoresistance of CRC cells through modulation of autophagy [23]. Although *Enterococcus faecalis* has been described as both a stimulant and a protector against CRC [50], recent studies have elucidated the role of biliverdin (i.e., one of its metabolites) as a tumor-stimulating compound that affects the host’s PI3K/AKT/mTOR pathway and thus promotes cell proliferation and angiogenesis [51,52]. Moreover, an experiment conducted by Chang et al. demonstrated that *Parvimonas micra* activated the Ras/ERK/c-Fos signaling pathway via micro-RNA upregulation and enhanced cellular proliferation in CRC [53]. Other studies have also highlighted *P. micra*’s implication in the immune response of CRC patients, potentially serving as a predictive biomarker for poor patient survival in CRC [54]. *Peptostreptococcus anaerobius* interacts directly with colonic cells via one of its surface proteins and also activates the proliferation-related integrin α2/β1-PI3K-Akt-NF-κB pathway [55]. Furthermore, *P. anaerobius* has been shown to intensify chemoresistance to oxaliplatin [56]. *Porphyromonas gingivalis* contributes to the proliferation of colorectal cancer cells through a mechanism involving cellular invasion and the subsequent activation of the MAPK/ERK signaling pathway [24]. Besides this role, a recent experiment showed that *P. gingivalis* upregulates chitinase 3-like-1 protein (CHI3L1) in invariant natural killer T (iNKT) cells, leading to detrimental effects on their cytotoxic function that result in the immune evasion of the tumors [57].

In parallel, recent studies highlight the importance of protein-level interactions between gut microbiota and the host. However, the experimental interactome remains limited, motivating the use of computational methods, including machine learning and domain-domain interaction prediction, to expand our understanding [32,33,34,35,36]. By integrating computational predictions with clinical microbiome data, this work contributes to mapping the molecular dialogs that underlie CRC development and potentially other microbiome-associated diseases.

The two phenotype-specific subnetworks, which were created after the categorization of the bacteria genera by phenotype association (i.e., health and CRC associated) after excluding the non-associated genera, revealed that the human proteins that are present in both subnetworks are the same. Interestingly, those human proteins also, when ranked according to their centrality degree, remain in the same order in both cases. This finding highlights not only the robustness of the human protein interactome and its key interactors but also emphasizes the complexity of the human proteins that can interact with different proteins at different health statuses. These most connected human proteins in these networks are mainly metalloproteins (E3 ubiquitin protein ligases, zinc transporters, etc.), transmembrane proteins, proteins related to ADP ribosylation and proteins related to mechanisms of cell proliferation and survival (i.e., apoptosis, programmed cell death, growth factors). It has been demonstrated that various species of pathogenic bacteria encode E3 ligases that have the ability to hijack the host’s ubiquitination mechanisms via a series of different strategies, including mimicking host-derived E3 ligases and encoding novel E3 ligases, as well as encoding deubiquitinases. Those proteins are transported into the host cell via type III or type IV secretion systems (T3SS and T4SS, respectively) and can then manipulate the host’s machinery for their proliferation [58,59]. Furthermore, there are studies that demonstrate the influence of the host’s zinc transporters on the homeostasis of the gut. A recent study showed that deletion of zinc (Zn) transporter ZIP14 creates a reduction in Zn in the entire intestinal tract and ultimately leads to lower microbial diversity [60,61]. Additionally, ATP-binding cassette (ABC) transporters have been associated with the microbial populations of the gut. More specifically, gut bacteria have been shown to both utilize them as binding receptors but also up- and downregulate their expression [62]. Research has also demonstrated that the bacterial ADP-ribosylation system, and mainly ADP-ribosyl transferases (ARTs), irreversibly modify host proteins with key function to the cellular cycle [63]. For example, Bxa of *Bacteroides* modifies non-muscle myosin II and triggers cellular remodeling that ultimately leads to inosine secretion that is then used by the microorganism as a carbon source [64]. Finally, since the gut bacteria have long been associated with cancer, mainly types of cancer that affect the GI tract. Therefore, it is no surprise that the gut microbiome influences growth factors, like the insulin-like growth factor 1 (IGF-1), which is essential for bone growth [65]. With respect to apoptosis, gut bacteria exert dual effects by either promoting or inhibiting cell death. Certain pathogens, such as non-typhoidal *Salmonella*, induce macrophage apoptosis via SPI-1 expression, thereby limiting inflammatory cytokine production [66]. Conversely, many bacterial species actively suppress host cell apoptosis to evade efferocytosis and enhance survival within the host [67].

On one hand, in the health-specific network, the most important proteins are those belonging to *Lachnospiraceae*, which are among the most abundant taxa in the GI tract. Evidence from multiple studies suggests that members of the *Lachnospiraceae* family contribute to maintaining the host’s physiological functions [68]. More specifically, they produce short-chain fatty acids that are converted into secondary bile acids that hinder the colonization of pathogenic strains [69].

On the other hand, in the bacteria-specific network, the most important proteins include several outer membrane, transport, and regulatory elements with distinct roles in microbial adaptation and survival. Among them, SusE and SusF are two outer membrane proteins from *Bacteroides* composed of tandem starch-specific carbohydrate-binding modules (CBMs) [70]. Although they lack enzymatic activity, these proteins are thought to play an essential role in starch metabolism by sequestering polysaccharides at the bacterial surface, thereby limiting access to competitor host cells [70]. Additional key proteins include alanine racemase, an enzyme required for the conversion of L-alanine to D-alanine and thus the synthesis of peptidoglycan, a critical component of bacterial cell walls [71], and ATP-binding cassette (ABC) transporters, which form a large superfamily of membrane complexes responsible for nutrient uptake, protein secretion, and resistance to environmental stressors, but also drug transfer [72,73]. Regulatory and DNA-binding proteins also contribute substantially to this network. These include lactose-binding proteins, such as the LacI repressor, which controls metabolic gene expression [74]. Enzymes such as thymidylate synthase, essential for deoxythymidine monophosphate (dTMP) synthesis [75], and adenine-specific DNA methyltransferases, which protect bacterial genomes from restriction enzymes [76], further emphasize the centrality of DNA-modifying activities. Finally, metalloproteins, particularly those containing iron–sulfur clusters, act as critical cofactors in electron transfer, catalysis, and gene regulation [77]. Collectively, these proteins reflect the molecular strategies employed by bacteria to secure nutrients, maintain genomic stability, and compete effectively within the intestinal ecosystem.

Apart from the most central proteins, proteins with centrality values near the mean also merit particular attention. From a graph-theoretical perspective, these nodes provide critical redundancy within the network: if only the most central proteins were perturbed, network function would collapse unless compensated by the surrounding intermediate nodes. Pathway enrichment analysis revealed that these moderately central proteins are implicated in the same biological pathways as the top-ranking hubs, thereby reinforcing their functional relevance. Their involvement suggests that they may act as auxiliary regulators or stabilizers, ensuring continuity of pathway activity and buffering the network against perturbations that target the primary hubs. Collectively, this highlights that both highly central and near-mean centrality proteins contribute to the robustness of bacteria–host interactions, and their combined roles are essential for maintaining network integrity under physiological and pathological conditions. The table with all the host and bacterial proteins with centrality values near the mean for both disease states can be found in Supplementary Materials File S4.

Interestingly, analysis of cross-distribution patterns revealed that CRC-associated taxa were also detectable within healthy samples, while health-associated taxa were observed in CRC samples. Quantitative analysis demonstrated that 48 bacterial taxa classified as CRC-associated were also detected in healthy samples, with an average relative abundance of approximately 0.27. Conversely, 40 taxa typically considered health-associated were found in CRC samples, with a higher mean abundance of about 1.03. These findings suggest that bacterial classification as CRC- or health-associated is not absolute but rather context-dependent, reflecting shifts in abundance and ecological balance rather than strict presence or absence. The observation that these taxa appear across both sample groups underscores the importance of relative abundance and community composition in shaping host–microbiome interactions and highlights that disease associations are likely driven by dysbiosis and altered network dynamics rather than by individual taxa alone. The tables with the Health-associated strains present in CRC patients and the CRC-associated strains in Healthy individuals, along with their relative abundances, can be found in Supplementary Materials Files S5 and S6.

Concerning the DL-based methodology, the integration of ProstT5-based embeddings and clinical metagenomics offers a high-resolution view of the CRC interactome, but it is essential to acknowledge the inherent constraints of a deep-learning-driven approach. The scientific rigor of this work is supported by the deliberate selection of primary, peer-reviewed repositories such as HPIDB, IntAct, and PHISTO for training, ensuring the model is grounded in the most comprehensive and experimentally validated interspecies data available. However, the dependence on high-fidelity input from predictive frameworks like AlphaFold and the “black-box” nature of deep neural networks present interpretability challenges regarding the precise biochemical drivers of each interaction. To mitigate the risks of dataset bias and overfitting common in high-dimensional biological spaces, we implemented technical safeguards, including focal loss functions and strict early stopping protocols. By prioritizing transparency in data filtering and calibrating thresholds to ensure a precision-driven interactome, this study provides a robust framework for identifying novel therapeutic targets and biomarkers while recognizing that these findings reflect computational predictions that require subsequent mechanistic validation. Finally, a limitation of this study is the lack of external validation in independent and ethnically diverse cohorts. While such validation is essential for assessing the generalizability of our findings, the present work was designed as a proof-of-concept study focusing on the development and internal evaluation of a deep learning framework for PPI mapping in a well-characterized colorectal cancer cohort from Crete/Greece. This setting provided a relatively homogeneous population, which facilitated controlled model development and reduced confounding variability during the initial phase of analysis. Future studies should aim to validate these findings across multicenter cohorts and diverse populations, as well as integrate additional omics datasets to further evaluate robustness and translational potential.

## 4. Materials and Methods

### 4.1. Training Dataset

This study began by compiling protein–protein interaction (PPI) data from multiple sources. Initially, we retrieved the pan-human–bacterial dataset comprising 19,686 experimentally validated interactions between 5714 bacterial and 4287 human proteins from four public databases: HPIDB [78,79], IntAct [80,81,82], PHISTO [83], and MorCVD [84]. To broaden our scope, we also incorporated a more inclusive PPI dataset from six widely used interaction databases (i.e., IntAct [80], MINT [85], DIP [86], HPRD [87], BioGRID [88], and SIFTS [89,90]). The selection of these specific repositories was based on their status as primary, peer-reviewed sources for experimentally validated interspecies interactions, ensuring the model was grounded in the most comprehensive and high-confidence data currently available in the field. This curated approach was designed to minimize noise and maximize the biological relevance of the training features. The dataset contained 1,081,401 PPIs, including 330,530 human inter-species PPIs and 750,871 inter- and intra-species interactions across diverse organisms. Notably, within this larger set, only 13 PPIs between host and gut bacterial proteins were identified, none involving proteoforms of the same gene.

To standardize structural information, all proteins from both datasets were mapped to their corresponding structures using the AlphaFold database API [91,92], mitigating potential biases from varying structural quality.

For constructing the positive dataset, both the original and larger PPI collections were filtered, retaining only interactions where both participating proteins had available structures. Conversely, the negative dataset, representing non-interacting pairs, was constructed using human proteins known to reside in different organs (data from Human Protein Atlas [93]) and whose domains (i.e., Pfam domains [94]) are not known to interact; the complete human proteome was sourced from UniProt Proteomes.

Furthermore, a gold-standard dataset of 17,278 experimentally supported domain-domain interactions (DDIs) from PDB complexes was retrieved from the 3did database [95]. These DDIs were then filtered to include only those not already present in our positive dataset or the known human interactome, ensuring unique, high-confidence examples.

The final curated dataset comprised a total of 16,690,098 PPI samples. To ensure robust model training and evaluation, these data were partitioned into training (60%—10,681,662 samples), validation (20%—2,670,416 samples), and test (20%—3,338,020 samples) subsets. A consistent class distribution was maintained across all subsets, reflecting the inherent imbalance of the interactome, with approximately 6% positive (interacting) and 94% negative (non-interacting) pairs. This distribution strategy was implemented to prevent overfitting and ensure that the performance metrics accurately reflect the model’s generalization capabilities on large-scale clinical data.

### 4.2. Deep Learning Model Architecture

The current study builds upon our previously published structure-based DL framework for human–gut bacterial PPI prediction [37]. While the architectural foundation remains unchanged, the protein feature extraction pipeline was modified to accommodate the unique demands of the current dataset and application domain. In the original model, protein structures were converted into graph representations and encoded using a pre-trained variational autoencoder (VAE). In this study, we replaced the VAE-based structural embedder with the Protein structure-sequence T5 (ProstT5) framework, a protein language model capable of capturing both sequential and structural information [96].

ProstT5 is a bilingual protein language model from the ProtT5 transformer architecture, fine-tuned to perform bi-directional translation between amino acid sequences and 3D structural representations. For the purposes of this study, we leveraged the encoder component of ProstT5 solely for protein feature extraction. Each protein sequence was converted into a fixed-size embedding vector using average pooling over the token-level embeddings obtained from ProstT5’s encoder. These embeddings were then used as inputs to the subsequent components of our established DL pipeline.

Unlike the structure-based MAPE-PPI encoder [97] used in prior work [37], which generates embeddings from a single processed representation of protein structure, ProstT5 leverages a bidirectional translation framework that learns jointly from amino acid sequences and structure-derived 3Di tokens. During pre-training, ProstT5 is trained not only to predict masked segments within either modality but also to translate from sequence to structure and from structure back to sequence, capturing the underlying correspondence between linear sequence patterns and their three-dimensional context. This bidirectional translation enables the model to internalize the mutual constraints between folding, residue interactions, and sequence evolution, producing embeddings that are structurally aware yet sequence-complete. At inference time, ProstT5 can generate informative embeddings directly from sequence data while implicitly encoding structural features, giving it a strong generalization advantage over models that depend on explicit sequence or structural inputs.

The downstream architecture, comprising a bi-directional Cross-Attention module and a fully connected interaction classifier, was preserved without modification. Briefly, the embeddings of a protein pair, i.e., one host and one and bacterial, were independently projected into a common subspace and fused via a multi-head cross-attention mechanism. This fusion step allows the model to capture inter-protein contextual dependencies and structural complementarity. The resulting interaction embedding was passed through a sequence of fully connected layers to produce a scalar interaction score. Class imbalance in the dataset was addressed using the focal loss function, and training was performed using the Adam optimizer with early stopping to prevent overfitting. Through this update, the model integrates ProstT5’s capacity to generalize across diverse protein sequences and structures while maintaining our previously validated attention-based interaction modeling and training strategy.

### 4.3. Application of DL Model on Clinical Data

#### 4.3.1. Clinical Data

**Figure 2:**
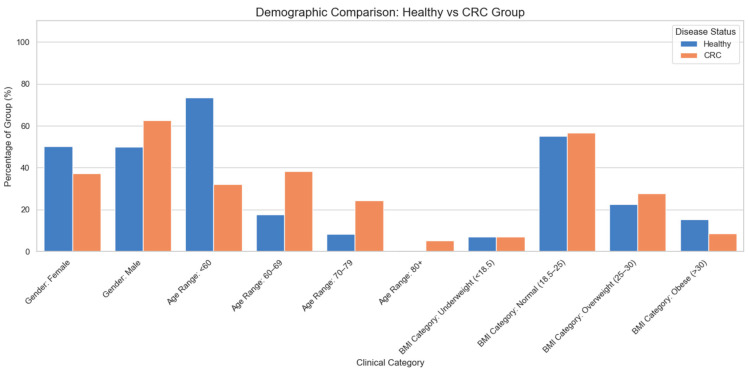
The demographic information of the clinical cohort.

The patient cohort (*N* = 152) comprised individuals diagnosed with colorectal cancer, collected at the time of initial diagnosis and before the application of any type of treatment. The patients were recruited from 3 major centers of colorectal surgery in Crete, Greece, thereby representing a geographically distinct population with inherent commonalities in dietary patterns, climatic exposure, and potentially other lifestyle factors. The control group (*N* = 91) consisted of people without any known pathology that could influence the homeostasis of the gut microbiome. The demographic information of the clinical cohort is presented in Figure 2. The 204 bacterial genera identified in both control and CRC samples were meticulously mapped to their respective strains. The primary resource for this mapping was the Human Gut Microbiome Atlas [98]. In cases where strain information was absent from the Atlas, data from UniProt [99,100] were employed for manual strain assignment. This detailed strain-level resolution was a necessary precursor to the subsequent computational investigations.

#### 4.3.2. Host–Microbiome PPI Network Prediction

Using the trained DL model, large-scale predictions of PPIs between human gut-expressed proteins and proteins derived from bacterial strains identified in each subject’s microbiome profile were performed. All pairwise combinations of host and microbial proteins were evaluated, and interaction probabilities were computed. The calibrated decision threshold, ensuring a minimum precision of 0.5 based on validation set performance, was applied to define interaction presence. The resulting subset of protein pairs was thus classified as interacting, forming an individual-level predicted interspecies PPI network. To further focus on the most confident predictions, a second filtering step was applied to the predicted network by retaining only protein pairs with posterior interaction probability close to 1.0.

#### 4.3.3. Post-Prediction Analysis of Interaction Probabilities

1. Mapping to Clinical Phenotypes: Each bacterial genus was annotated based on the disease status of the individual (colorectal cancer vs. healthy control), facilitating a group-specific comparison of PPI patterns. Non-parametric statistical testing at the taxonomic level was performed to detect significant differences in relative abundance between the two phenotypic groups. For each taxon, a Mann–Whitney U test (two-sided) was conducted to compare abundance distributions between healthy and cancer samples, selected for its robustness to non-normal distributions and unequal variances [101]. The median abundance in each phenotypic group, the Mann–Whitney U statistic, and the associated *p*-value were computed. For multiple hypothesis testing correction, the Benjamini–Hochberg procedure was applied to control the false discovery rate (FDR), reporting q-values alongside significance indicators (q-value < 0.05) [102]. Each genus was classified based on its differential abundance profile. Taxa with statistically significant differences (FDR-corrected q-value < 0.05) were labeled as either:
  - CRC-associated: higher median abundance in the CRC cohort.
  - Health-associated: higher median abundance in the healthy cohort.

Samples without significant differences, or with identical medians across groups, were labeled as Both (No clear association).

2. Aggregation at the Genus Level: To generalize interaction patterns across microbial taxonomy, predicted interactions were aggregated per bacterial genus.
3. Comparative Analysis by Disease State: Identification of genera or host targets with distinct interaction profiles in cancer versus health state.

#### 4.3.4. Topological Analysis

The resulting high-confidence network was subjected to graph theory analysis. Degree centrality was computed to identify central nodes within the host and bacterial sub-networks, highlighting putative key mediators of host–microbiome molecular interaction. All bacterial proteins were remapped to their corresponding genus and disease state to facilitate downstream comparative analyses.

#### 4.3.5. Co-Occurrence Network Construction

In parallel, co-occurrence networks of bacterial genera were constructed separately for the healthy and CRC cohorts. Pairwise Pearson correlation coefficients were calculated between genus-level abundances across samples. Only statistically significant and strong associations (|r| > 0.6, *p* < 0.05) were retained, resulting in genus–genus networks representing co-existence patterns. These networks were analyzed using degree centrality, and community detection was also applied to identify microbiome clusters.

#### 4.3.6. Comparative Analysis of Co-Occurrence and Predicted Molecular Networks

To explore convergence between co-occurrence and molecular interaction patterns, an examination was conducted to determine whether bacterial genera co-occurring in the ecological network also exhibited shared or functionally similar PPI profiles with the human host. Bacterial proteomes from the co-occurrence networks were cross-referenced with those used in the DL-based predictions. Further filtering was applied to ensure taxonomic consistency, excluding unmatched strains. Similarity of host target profiles was assessed among co-occurring genera to evaluate potential functional redundancy or cooperative behavior.

#### 4.3.7. Functional Characterization of Host Targets

Finally, human proteins identified as central nodes in the predicted PPI network were subjected to functional enrichment analysis to determine their involvement in cancer-related processes such as inflammation, immune modulation, apoptosis, and metabolism. This analysis was further contextualized by mapping bacterial network clusters (derived from co-occurrence patterns) to their corresponding host interaction signatures.

## 5. Conclusions

This study integrates clinical microbiome data with a deep learning framework for host–microbe protein–protein interaction (PPI) prediction to provide new insights into the molecular basis of colorectal cancer (CRC). By mapping phenotype-specific PPI networks, we identified highly central bacterial and human proteins that may serve as key mediators of host–microbiome crosstalk. Interestingly, human proteins maintained consistent centrality across both healthy- and CRC-associated networks, underscoring their robustness and pivotal roles in cellular processes such as ubiquitination, apoptosis, zinc transport, and growth regulation. On the microbial side, central proteins highlighted distinct strategies of adaptation and survival, ranging from starch sequestration and ABC transporter activity to DNA modification and electron transfer.

Beyond the most central proteins, nodes with near-mean centrality were shown to contribute substantially to network resilience, participating in the same pathways as the hubs and acting as auxiliary stabilizers of host–microbiome interactions. Therefore, CRC pathogenicity cannot be addressed solely by targeting hubs but requires intervention across the entire pathway that secondary nodes stabilize. Furthermore, cross-distribution analyses revealed that CRC-associated taxa were present in healthy samples, and health-associated taxa were detectable in CRC, indicating that disease associations are more strongly linked to dysbiosis and ecological imbalance than to strict taxonomic exclusivity.

Collectively, these findings reinforce the concept that colorectal cancer arises not from the influence of single bacterial species alone but from complex, network-level perturbations in host–microbiome interactions. By demonstrating the utility of computational PPI prediction in conjunction with clinical microbiome profiling, this work provides a framework for uncovering mechanistic pathways of CRC development and opens avenues for the identification of novel therapeutic targets and biomarkers.

## Figures and Tables

**Table 1:** Evaluation metrics for prediction on the test portion of the PPI dataset.

Precision (macro)	0.9911
Recall (macro)	0.9747
F1 (macro)	0.9827
Precision (positive)	0.9855
Recall (positive)	0.9503
F1 (Positive)	0.9676
MCC	0.9657
Balanced Accuracy	0.9747
AP	0.9867
AUROC	0.9959
Recall@precision_50	0.9901

**Table 2:** Confusion Matrix for the prediction on the test portion of the PPI dataset.

	Predicted Negative	Predicted Positive
Actual Negative	3,129,949	2853
Actual Positive	10,133	194,112

**Table 3:** Phenotype association information.

Phenotype Association	Number of Samples
Both (No clear association)	116
CRC-associated	48
Health-associated	40

**Table 4:** CRC-associated samples.

Taxonomy	Median	*p*-Value	q-Value
g__unknown_Finegoldia	0.9480	1.472 × 10^−39^	1.502 × 10^−37^
g__unknown_Anaerococcus	0.5784	1.238 × 10^−39^	1.502 × 10^−37^
g__unknown_Peptoniphilus	1.2181	3.501 × 10^−39^	2.381 × 10^−37^
g__Corynebacterium	0.0759	1.440 × 10^−34^	6.923 × 10^−33^
g__Porphyromonas	0.4821	1.697 × 10^−34^	6.923 × 10^−33^
g__unknown_Ezakiella	0.1024	1.342 × 10^−31^	4.562 × 10^−30^
g__Campylobacter	0.1419	2.343 × 10^−31^	6.828 × 10^−30^
g__Lawsonella	0.0139	5.945 × 10^−31^	1.516 × 10^−29^
g__S5-A14a	0.0719	8.633 × 10^−30^	1.957 × 10^−28^
g__Fusobacterium	0.0626	3.299 × 10^−28^	6.729 × 10^−27^
g__unknown_Fenollaria	0.1280	1.485 × 10^−27^	2.753 × 10^−26^
g__Mobiluncus	0.0107	4.018 × 10^−26^	6.306 × 10^−25^
g__unknown_Murdochiella	0.0497	4.575 × 10^−25^	6.222 × 10^−24^
g__Peptostreptococcus	0.0376	4.423 × 10^−25^	6.222 × 10^−24^
g__Varibaculum	0.0053	8.724 × 10^−24^	1.112 × 10^−22^
g__Peptococcus	0.0184	4.041 × 10^−23^	4.849 × 10^−22^
g__unknown_Fastidiosipila	0.0073	5.704 × 10^−23^	6.464 × 10^−22^
g__Pyramidobacter	0.0034	2.621 × 10^−20^	2.814 × 10^−19^
g__unknown_Parvimonas	0.0156	3.295 × 10^−20^	3.361 × 10^−19^
g__Negativicoccus	0.0024	1.407 × 10^−19^	1.367 × 10^−18^
g__Escherichia-Shigella	0.6294	1.608 × 10^−18^	1.491 × 10^−17^
g__Lactobacillus	0.0120	6.432 × 10^−17^	5.652 × 10^−16^
g__unknown_Gallicola	0.0015	6.650 × 10^−17^	5.652 × 10^−16^
g__Facklamia	0.0007	1.215 × 10^−15^	9.911 × 10^−15^
g__Enterococcus	0.0298	1.829 × 10^−15^	1.435 × 10^−14^
g__Gemella	0.0047	3.007 × 10^−15^	2.272 × 10^−14^
g__Streptococcus	0.7263	2.605 × 10^−13^	1.715 × 10^−12^
g__Negativibacillus	0.1024	2.994 × 10^−12^	1.745 × 10^−11^
g__Prevotella	1.9324	2.928 × 10^−11^	1.615 × 10^−10^
g__Staphylococcus	0.0062	8.023 × 10^−11^	4.307 × 10^−10^
g__Actinomyces	0.0103	3.922 × 10^−10^	2.000 × 10^−9^
g__Solobacterium	0.0016	6.767 × 10^−9^	3.068 × 10^−8^
g__*Ruminococcus torques* group	1.2661	2.016 × 10^−8^	7.758 × 10^−8^
g__Methanobrevibacter	0.0003	2.611 × 10^−8^	9.863 × 10^−8^
g__Klebsiella	0.0051	1.352 × 10^−7^	4.924 × 10^−7^
g__unknown_Atopobiaceae	0.0013	1.624 × 10^−7^	5.812 × 10^−7^
g__Blautia	4.5043	5.155 × 10^−7^	1.724 × 10^−6^
g__Sellimonas	0.0195	6.426 × 10^−7^	2.081 × 10^−6^
g__*Clostridium innocuum* group	0.0064	1.110 × 10^−6^	3.538 × 10^−6^
g__unknown_Peptostreptococcaceae	0.2428	2.851 × 10^−6^	8.681 × 10^−6^
g__Dorea	0.2093	3.859 × 10^−6^	1.158 × 10^−5^
g__Collinsella	0.5357	5.222 × 10^−6^	1.522 × 10^−5^
g__*Clostridium sensu* stricto 1	0.1232	1.408 × 10^−5^	3.729 × 10^−5^
g__Desulfovibrio	0.0036	4.232 × 10^−5^	1.066 × 10^−4^
g__Slackia	0.0014	1.788 × 10^−3^	3.286 × 10^−3^
g__Anaerotruncus	0.0040	1.480 × 10^−2^	2.305 × 10^−2^
g__unknown_Murib.302aculaceae	0.0044	1.561 × 10^−2^	2.395 × 10^−2^
g__Paraprevotella	0.0008	2.506 × 10^−2^	3.704 × 10^−2^

**Table 5:** Healthy-associated samples.

Taxonomy	Median	*p*-Value	q-Value
g__*Eubacterium eligens* group	1.9933	3.020 × 10^−26^	5.134 × 10^−25^
g__Monoglobus	0.2625	6.650 × 10^−15^	4.845 × 10^−14^
g__Faecalibacterium	9.5803	4.137 × 10^−14^	2.910 × 10^−13^
g__Lachnospiraceae NK4A136 group	2.4790	1.731 × 10^−13^	1.177 × 10^−12^
g__UCG-003	0.3445	8.407 × 10^−13^	5.197 × 10^−12^
g__GCA-900066575	0.0446	1.344 × 10^−12^	8.064 × 10^−12^
g__unknown_Ruminococcaceae	0.1930	1.042 × 10^−11^	5.902 × 10^−11^
g__Subdoligranulum	2.9305	3.706 × 10^−10^	1.938 × 10^−9^
g__Alistipes	2.0103	3.612 × 10^−9^	1.714 × 10^−8^
g__Coprobacter	0.0174	8.986 × 10^−9^	3.819 × 10^−8^
g__Colidextribacter	0.1649	1.162 × 10^−8^	4.838 × 10^−8^
g__*Eubacterium ventriosum* group	0.1361	1.443 × 10^−8^	5.888 × 10^−8^
g__DTU089	0.0082	1.874 × 10^−8^	7.444 × 10^−8^
g__Christensenellaceae R-7 group	0.7801	2.074 × 10^−7^	7.296 × 10^−7^
g__Adlercreutzia	0.0540	3.301 × 10^−7^	1.141 × 10^−6^
g__unknown_Clostridia UCG-014	0.7140	4.265 × 10^−7^	1.450 × 10^−6^
g__Bacteroides	21.5657	5.576 × 10^−7^	1.835 × 10^−6^
g__Oscillospira	0.0227	2.372 × 10^−6^	7.331 × 10^−6^
g__Fusicatenibacter	1.8678	4.795 × 10^−6^	1.418 × 10^−5^
g__Haemophilus	0.0209	6.277 × 10^−6^	1.778 × 10^−5^
g__Oscillibacter	0.1694	6.731 × 10^−6^	1.873 × 10^−5^
g__Anaerostipes	0.3767	6.794 × 10^−6^	1.873 × 10^−5^
g__Erysipelotrichaceae UCG-003	0.2423	1.202 × 10^−5^	3.226 × 10^−5^
g__Bifidobacterium	0.8648	1.901 × 10^−5^	4.971 × 10^−5^
g__Butyricicoccus	0.3858	2.780 × 10^−5^	7.088 × 10^−5^
g__UCG-002	1.9335	5.882 × 10^−5^	1.463 × 10^−4^
g__unknown_Lachnospiraceae	6.9246	6.741 × 10^−5^	1.637 × 10^−4^
g__UBA1819	0.0199	1.925 × 10^−4^	4.411 × 10^−4^
g__Veillonella	0.0205	3.616 × 10^−4^	7.683 × 10^−4^
g__Barnesiella	0.6569	5.741 × 10^−4^	1.195 × 10^−3^
g__NK4A214 group	0.3200	1.046 × 10^−3^	2.072 × 10^−3^
g__unknown_Clostridia vadinBB60 group	0.0448	1.087 × 10^−3^	2.132 × 10^−3^
g__UCG-005	0.4769	1.141 × 10^−3^	2.197 × 10^−3^
g__Roseburia	0.0164	1.355 × 10^−3^	2.536 × 10^−3^
g__Sutterella	0.9726	2.152 × 10^−3^	3.785 × 10^−3^
g__UCG-009	0.0058	6.594 × 10^−3^	1.112 × 10^−2^
g__Coprococcus	0.9442	1.291 × 10^−2^	2.058 × 10^−2^
g__*Eubacterium siraeum* group	0.1397	1.386 × 10^−2^	2.174 × 10^−2^
g__Parasutterella	0.0600	1.505 × 10^−2^	2.325 × 10^−2^
g__Lachnospiraceae ND3007 group	0.2239	1.706 × 10^−2^	2.577 × 10^−2^

**Table 6:** Top-20 Degree: Health-associated interactions.

Protein Type	Uniprot ID	Degree	Name	Species
Bacterial Proteins	A0A1T5GC38	29,343	Cellulase (Glycosyl hydrolase family 5)	*Lachnospiraceae bacterium*
	A0A1T5D2V2	29,307	Sugar phosphate isomerase/epimerase	*Lachnospiraceae bacterium*
	A0A1T5GAW1	29,307	Thymidylate synthase (EC 2.1.1.45)	*Lachnospiraceae bacterium*
	A0A1T5BRU8	29,307	ATPase/GTPase, AAA15 family	*Lachnospiraceae bacterium*
	A0A1T5CV36	29,280	GTP cyclohydrolase 1 type 2 homolog	*Lachnospiraceae bacterium*
	A0A1T5CWM9	29,259	HPr Serine kinase C-terminal domain-containing protein	*Lachnospiraceae bacterium*
	A0A1T5EZK8	29,259	ATPase/GTPase, AAA15 family	*Lachnospiraceae bacterium*
	A0A1T5FSD0	29,226	Extracellular solute-binding protein	*Lachnospiraceae bacterium*
	A0A1T5BTE9	29,226	Sugar phosphate isomerase/epimerase	*Lachnospiraceae bacterium*
	A0A1T5D3P2	29,097	ATPase/GTPase, AAA15 family	*Lachnospiraceae bacterium*
	A0A1T5FKU6	29,094	M6 family metalloprotease domain-containing protein	*Lachnospiraceae bacterium*
	A0A1T5FSP8	29,085	Predicted dehydrogenase	*Lachnospiraceae bacterium*
	A0A1T5FSV4	29,079	Sugar phosphate isomerase/epimerase	*Lachnospiraceae bacterium*
	A0A1T5C7W1	28,980	L-ribulose-5-phosphate 3-epimerase	*Lachnospiraceae bacterium*
	A0A1T5EFX0	28,974	Condensation domain-containing protein	*Lachnospiraceae bacterium*
	A0A1T5E9H3	28,854	Ribose transport system permease protein	*Lachnospiraceae bacterium*
	A0A1T5CBP8	28,836	Autoinducer 2 import system permease protein LsrD	*Lachnospiraceae bacterium*
	A0A1T5G1V4	28,830	Putative selenium metabolism hydrolase	*Lachnospiraceae bacterium*
	A0A1T5FWX8	28,830	Putative peptidoglycan binding domain-containing protein	*Lachnospiraceae bacterium*
	A0A1T5FKR2	28,785	D-alanyl-D-alanine carboxypeptidase (Penicillin-binding protein 5/6)	*Lachnospiraceae bacterium*
Human Proteins	O00165	693,752	HCLS1-associated protein X-1 (HS1-associating protein X-1) (HAX-1) (HS1-binding protein 1) (HSP1BP-1)	*Homo sapiens* (Human)
	O15105	664,028	Mothers against decapentaplegic homolog 7 (MAD homolog 7) (Mothers against DPP homolog 7) (Mothers against decapentaplegic homolog 8) (MAD homolog 8) (Mothers against DPP homolog 8) (SMAD family member 7) (SMAD 7) (Smad7) (hSMAD7)	*Homo sapiens* (Human)
	Q92504	634,221	Zinc transporter SLC39A7 (Histidine-rich membrane protein Ke4) (Really interesting new gene 5 protein) (Solute carrier family 39 member 7) (Zrt-, Irt-like protein 7) (ZIP7)	*Homo sapiens* (Human)
	Q6E0U4	626921	Dermokine (Epidermis-specific secreted protein SK30/SK89)	*Homo sapiens* (Human)
	P54826	614,560	Growth arrest-specific protein 1 (GAS-1)	*Homo sapiens* (Human)
	Q9HC07	599,200	Putative divalent cation/proton antiporter TMEM165 (Transmembrane protein 165) (Transmembrane protein PT27) (Transmembrane protein TPARL)	*Homo sapiens* (Human)
	Q9NVW2	586,421	E3 ubiquitin-protein ligase RLIM (EC 2.3.2.27) (LIM domain-interacting RING finger protein) (RING finger LIM domain-binding protein) (R-LIM) (RING finger protein 12) (RING-type E3 ubiquitin transferase RLIM) (Renal carcinoma antigen NY-REN-43)	*Homo sapiens* (Human)
	Q15773	586,010	Myeloid leukemia factor 2 (Myelodysplasia-myeloid leukemia factor 2)	*Homo sapiens* (Human)
	Q9Y252	581,832	E3 ubiquitin-protein ligase RNF6 (EC 2.3.2.27)	*Homo sapiens* (Human)
	Q9BZR8	576,099	Apoptosis facilitator Bcl-2-like protein 14 (Bcl2-L-14) (Apoptosis regulator Bcl-G)	*Homo sapiens* (Human)
	Q9UHA4	552,657	Ragulator complex protein LAMTOR3 (Late endosomal/lysosomal adaptor and MAPK and MTOR activator 3) (MEK-binding partner 1) (Mp1) (Mitogen-activated protein kinase 1-interacting protein 1) (Mitogen-activated protein kinase scaffold protein 1)	*Homo sapiens* (Human)
	Q9BRP1	535,201	Programmed cell death protein 2-like	*Homo sapiens* (Human)
	P62993	535,073	Growth factor receptor-bound protein 2 (Adapter protein GRB2) (Protein Ash) (SH2/SH3 adapter GRB2)	*Homo sapiens* (Human)
	Q969M3	531,043	Protein YIPF5 (Five-pass transmembrane protein localizing in the Golgi apparatus and the endoplasmic reticulum 5) (Smooth muscle cell-associated protein 5) (SMAP-5) (YIP1 family member 5) (YPT-interacting protein 1 A)	*Homo sapiens* (Human)
	Q9Y4L5	528,189	E3 ubiquitin-protein ligase RNF115 (EC 2.3.2.27) (RING finger protein 115) (RING-type E3 ubiquitin transferase RNF115) (Rab7-interacting RING finger protein) (Rabring 7) (Zinc finger protein 364)	*Homo sapiens* (Human)
	Q8N6T3	517,063	ADP-ribosylation factor GTPase-activating protein 1 (ARF GAP 1) (ADP-ribosylation factor 1 GTPase-activating protein) (ARF1 GAP) (ARF1-directed GTPase-activating protein)	*Homo sapiens* (Human)
	Q9BW91	512,704	ADP-ribose pyrophosphatase, mitochondrial (EC 3.6.1.13) (ADP-ribose diphosphatase) (ADP-ribose phosphohydrolase) (Adenosine diphosphoribose pyrophosphatase) (ADPR-PPase) (Nucleoside diphosphate-linked moiety X motif 9) (Nudix motif 9)	*Homo sapiens* (Human)
	Q02535	511,921	DNA-binding protein inhibitor ID-3 (Class B basic helix-loop-helix protein 25) (bHLHb25) (Helix-loop-helix protein HEIR-1) (ID-like protein inhibitor HLH 1R21) (Inhibitor of DNA binding 3) (Inhibitor of differentiation 3)	*Homo sapiens* (Human)
	Q9H0V1	511,224	Transmembrane protein 168	*Homo sapiens* (Human)
	Q9NZ45	510,746	CDGSH iron-sulfur domain-containing protein 1 (Cysteine transaminase CISD1) (EC 2.6.1.3) (MitoNEET)	*Homo sapiens* (Human)

**Table 7:** Top-20 Degree: CRC-associated interactions.

Protein Type	Uniprot ID	Degree	Name	Species
Bacterial Proteins	R6WAI1	19,562	ABC-type transport system substrat-binding component	*Ruminococcus* sp. CAG:382
	R6XB82	19,562	Outer membrane protein SusF	*Prevotella* sp. CAG:732
	S0IX76	19,556	Solute-binding protein family 5 domain-containing protein	*Eubacterium* sp. 14-2
	R6XFW8	19,556	Putative cellulase	*Prevotella* sp. CAG:732
	Q8RG28	19,556	Alanine racemase (EC 5.1.1.1)	*Fusobacterium nucleatum* subsp. nucleatum (strain ATCC 25586/DSM 15643/BCRC 10681/CIP 101130/JCM 8532/KCTC 2640/LMG 13131/VPI 4355)
	A0A173W5P6	19,556	Lactose-binding protein	[Ruminococcus] torques
	R7KUC7	19,556	ABC transporter solute-binding protein	*Ruminococcus* sp. CAG:353
	Q8REC7	19,554	Type I-B CRISPR-associated protein Cas7/Cst2/DevR	*Fusobacterium nucleatum* subsp. nucleatum (strain ATCC 25586/DSM 15643/BCRC 10681/CIP 101130/JCM 8532/KCTC 2640/LMG 13131/VPI 4355)
	R6Q3S4	19,554	Outer membrane protein SusF/SusE-like C-terminal domain-containing protein	*Prevotella* sp. CAG:386
	R6XJ01	19,548	Concanavalin A-like lectin/glucanases family protein	*Prevotella* sp. CAG:732
	R5LV05	19,548	Outer membrane protein SusE	*Prevotella* sp. CAG:1185
	R6XA89	19,548	Putative iron-sulfur cluster-binding protein	*Ruminococcus* sp. CAG:382
	R6W4K0	19,546	Carbohydrate-binding domain-containing protein	*Ruminococcus* sp. CAG:382
	R6F9J0	19,542	Right-handed beta helix domain-containing protein	*Prevotella* sp. CAG:520
	A0A239RL25	19,542	Thymidylate synthase	*Prevotellaceae bacterium* KH2P17
	R5PWH5	19,542	site-specific DNA-methyltransferase (adenine-specific) (EC 2.1.1.72)	*Prevotella* sp. CAG:1092
	R7KSV1	19,542	Glycoside hydrolase family 16	*Ruminococcus* sp. CAG:353
	R6W021	19,542	Oligopeptide ABC superfamily ATP binding cassette transporter binding protein	*Ruminococcus* sp. CAG:382
	R5PW27	19,540	Lipoprotein	*Prevotella* sp. CAG:1092
	R6EGC3	19,540	Concanavalin A-like lectin/glucanases family protein	*Prevotella* sp. CAG:1320
Human Proteins	O00165	618,870	HCLS1-associated protein X-1 (HS1-associating protein X-1) (HAX-1) (HS1-binding protein 1) (HSP1BP-1)	*Homo sapiens* (Human)
	O15105	591,247	Mothers against decapentaplegic homolog 7 (MAD homolog 7) (Mothers against DPP homolog 7) (Mothers against decapentaplegic homolog 8) (MAD homolog 8) (Mothers against DPP homolog 8) (SMAD family member 7) (SMAD 7) (Smad7) (hSMAD7)	*Homo sapiens* (Human)
	Q92504	563,786	Zinc transporter SLC39A7 (Histidine-rich membrane protein Ke4) (Really interesting new gene 5 protein) (Solute carrier family 39 member 7) (Zrt-, Irt-like protein 7) (ZIP7)	*Homo sapiens* (Human)
	Q6E0U4	557,079	Dermokine (Epidermis-specific secreted protein SK30/SK89)	*Homo sapiens* (Human)
	P54826	545,772	Growth arrest-specific protein 1 (GAS-1)	*Homo sapiens* (Human)
	Q9HC07	531,560	Putative divalent cation/proton antiporter TMEM165 (Transmembrane protein 165) (Transmembrane protein PT27) (Transmembrane protein TPARL)	*Homo sapiens* (Human)
	Q9NVW2	519,970	E3 ubiquitin-protein ligase RLIM (EC 2.3.2.27) (LIM domain-interacting RING finger protein) (RING finger LIM domain-binding protein) (R-LIM) (RING finger protein 12) (RING-type E3 ubiquitin transferase RLIM) (Renal carcinoma antigen NY-REN-43)	*Homo sapiens* (Human)
	Q15773	519,551	Myeloid leukemia factor 2 (Myelodysplasia-myeloid leukemia factor 2)	*Homo sapiens* (Human)
	Q9Y252	515,797	E3 ubiquitin-protein ligase RNF6 (EC 2.3.2.27)	*Homo sapiens* (Human)
	Q9BZR8	510,681	Apoptosis facilitator Bcl-2-like protein 14 (Bcl2-L-14) (Apoptosis regulator Bcl-G)	*Homo sapiens* (Human)
	Q9UHA4	489,358	Ragulator complex protein LAMTOR3 (Late endosomal/lysosomal adaptor and MAPK and MTOR activator 3) (MEK-binding partner 1) (Mp1) (Mitogen-activated protein kinase 1-interacting protein 1) (Mitogen-activated protein kinase scaffold protein 1)	*Homo sapiens* (Human)
	Q9BRP1	473,519	Programmed cell death protein 2-like	*Homo sapiens* (Human)
	P62993	473,461	Growth factor receptor-bound protein 2 (Adapter protein GRB2) (Protein Ash) (SH2/SH3 adapter GRB2)	*Homo sapiens* (Human)
	Q969M3	469,851	Protein YIPF5 (Five-pass transmembrane protein localizing in the Golgi apparatus and the endoplasmic reticulum 5) (Smooth muscle cell-associated protein 5) (SMAP-5) (YIP1 family member 5) (YPT-interacting protein 1 A)	*Homo sapiens* (Human)
	Q9Y4L5	467,166	E3 ubiquitin-protein ligase RNF115 (EC 2.3.2.27) (RING finger protein 115) (RING-type E3 ubiquitin transferase RNF115) (Rab7-interacting RING finger protein) (Rabring 7) (Zinc finger protein 364)	*Homo sapiens* (Human)
	Q8N6T3	457,183	ADP-ribosylation factor GTPase-activating protein 1 (ARF GAP 1) (ADP-ribosylation factor 1 GTPase-activating protein) (ARF1 GAP) (ARF1-directed GTPase-activating protein)	*Homo sapiens* (Human)
	Q9BW91	453,329	ADP-ribose pyrophosphatase, mitochondrial (EC 3.6.1.13) (ADP-ribose diphosphatase) (ADP-ribose phosphohydrolase) (Adenosine diphosphoribose pyrophosphatase) (ADPR-PPase) (Nucleoside diphosphate-linked moiety X motif 9) (Nudix motif 9)	*Homo sapiens* (Human)
	Q02535	452,521	DNA-binding protein inhibitor ID-3 (Class B basic helix-loop-helix protein 25) (bHLHb25) (Helix-loop-helix protein HEIR-1) (ID-like protein inhibitor HLH 1R21) (Inhibitor of DNA binding 3) (Inhibitor of differentiation 3)	*Homo sapiens* (Human)
	Q9H0V1	452,025	Transmembrane protein 168	*Homo sapiens* (Human)
	Q9NZ45	451,580	CDGSH iron-sulfur domain-containing protein 1 (Cysteine transaminase CISD1) (EC 2.6.1.3) (MitoNEET)	*Homo sapiens* (Human)

## Data Availability

The original contributions presented in this study are included in the article/Supplementary Materials. Further inquiries can be directed to the corresponding author.

## Supplementary Materials

The following supporting information can be downloaded at: https://www.mdpi.com/article/10.3390/ijms27104232/s1.
